# New Approaches for the Treatment of Chronic Graft-Versus-Host Disease: Current Status and Future Directions

**DOI:** 10.3389/fimmu.2020.578314

**Published:** 2020-10-09

**Authors:** Nathaniel Edward Bennett Saidu, Chiara Bonini, Anne Dickinson, Magdalena Grce, Marit Inngjerdingen, Ulrike Koehl, Antoine Toubert, Robert Zeiser, Sara Galimberti

**Affiliations:** ^1^Division of Molecular Medicine, Ruđer Bošković Institute, Zagreb, Croatia; ^2^Department of Pharmacology, University of Oslo and Oslo University Hospital, Oslo, Norway; ^3^Experimental Hematology Unit, San Raffaele Scientific Institute, Milano, Italy; ^4^Haematological Sciences, Newcastle University, Newcastle upon Tyne, United Kingdom; ^5^Faculty of Medicine, Institute of Clinical Immunology, University Leipzig and Fraunhofer IZI, Leipzig, Germany; ^6^Université de Paris, Institut de Recherche Saint Louis, EMiLy, Inserm U1160, Paris, France; ^7^Laboratoire d'Immunologie et d`Histocompatibilité, AP-HP, Hopital Saint-Louis, Paris, France; ^8^Department of Hematology, Oncology and Stem Cell Transplantation, Freiburg University Medical Center, Faculty of Medicine, Freiburg, Germany; ^9^Department of Clinical and Experimental Medicine, University of Pisa, Pisa, Italy

**Keywords:** chronic graft-versus-host disease, tyrosine kinase inhibitors, immunotherapy, Janus kinase 1/2, hematopoietic stem cell transplantation

## Abstract

Chronic graft-versus-host disease (cGvHD) is a severe complication of allogeneic hematopoietic stem cell transplantation that affects various organs leading to a reduced quality of life. The condition often requires enduring immunosuppressive therapy, which can also lead to the development of severe side effects. Several approaches including small molecule inhibitors, antibodies, cytokines, and cellular therapies are now being developed for the treatment of cGvHD, and some of these therapies have been or are currently tested in clinical trials. In this review, we discuss these emerging therapies with particular emphasis on tyrosine kinase inhibitors (TKIs). TKIs are a class of compounds that inhibits tyrosine kinases, thereby preventing the dissemination of growth signals and activation of key cellular proteins that are involved in cell growth and division. Because they have been shown to inhibit key kinases in both B cells and T cells that are involved in the pathophysiology of cGvHD, TKIs present new promising therapeutic approaches. Ibrutinib, a Bruton tyrosine kinase (Btk) inhibitor, has recently been approved by the Food and Drug Administration (FDA) in the United States for the treatment of adult patients with cGvHD after failure of first-line of systemic therapy. Also, Janus Associated Kinases (JAK1 and JAK2) inhibitors, such as itacitinib (JAK1) and ruxolitinib (JAK1 and 2), are promising in the treatment of cGvHD. Herein, we present the current status and future directions of the use of these new drugs with particular spotlight on their targeting of specific intracellular signal transduction cascades important for cGvHD, in order to shed some light on their possible mode of actions.

## Introduction

GvHD is a complication that often occurs after allogeneic hematopoietic stem cell transplantation (allo-HSCT) in up to 50% of cases, where donor T- and B cells derived from the graft recognize and attack host antigens (1). This is particularly important because HSCT is the treatment of choice for many types of malignant and non-malignant hematological and autoimmune diseases. According to the onset, GvHD can be acute (aGvHD) or chronic (cGvHD): aGvHD typically occurs in less than 100 days after transplantation and it is associated with inflammation in several organs or sites, such as skin, gastrointestinal tract, mouth, genital tract or liver, while cGvHD may present at any time after HSCT, even if it typically occurs after 100 days from graft infusion (2).

Thus, from the clinical point of view, cGvHD resembles an “autoimmune syndrome”, characterized by immune dysregulation and absence of functional tolerance. As in systemic rheumatologic diseases, its clinical manifestations are variable, varying from lichen planus-like lesions to full sclerosis, muscle pain or joint fasciitis, vulvo-vaginitis, bronchiolitis obliterans (BO), in addition to damage of gastrointestinal tract and liver (3).

The National Institute of Health (NIH) consensus established diagnostic criteria for cGvHD in 2005 (4), which were revised in 2014 (5). The authors defined cGvHD-specific organ manifestations and elaborated a scoring system by considering the severity of involvement of skin, mouth, eyes, gastrointestinal tract, liver, lungs, joint fascia, and genital tract differently from other hematological diseases, and from models for testing conditioning or immunosuppressive regimens. Unfortunately, the existing murine models of cGvHD fail to fully include the whole spectrum of human cGvHD symptoms, which makes it difficult to treat and to draw general conclusions about the efficacy of new therapeutic approaches based on animal models (6).

**Figure 1:**
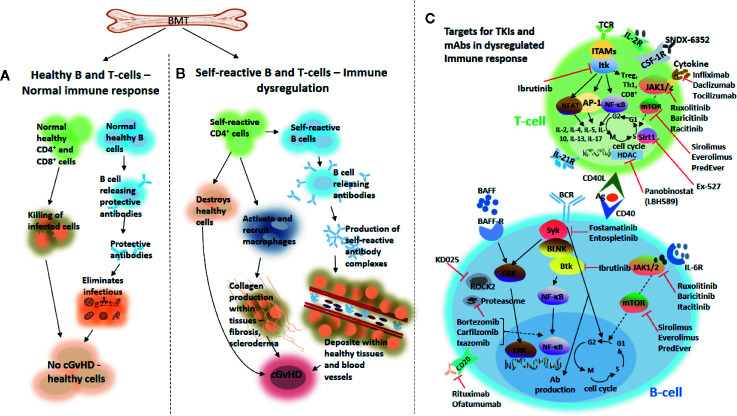
Chronic GvHD development and novel agents targeting B and T cells that are under investigation for the treatment of the disease. Following bone marrow transplantation, healthy production of effector B and T cells from the bone marrow may trigger a normal healthy immune response leading to a healthy immune homeostasis **(A)**. Overproduction of self-reactive B and T cells from donor-derived bone marrow grafts may cause immune dysregulation, which on the one hand may lead to the destruction of healthy tissues, activate and recruit macrophages important for the production of collagen within tissues, thereby, causing fibrosis and scleroderma and subsequently, development of cGvHD. On the other hand, production of self-reactive antibody complexes may be triggered by self-reactive B cells from donor-derived bone marrow grafts, which may be deposited into healthy tissues and blood vessels and subsequently leading to the development of cGvHD **(B)**. Novel agents targeting either B- or T cells that are under investigation for the treatment of cGvHD **(C)**. TCR, T cell receptor; TKIs, tyrosine kinase inhibitors; IL-2R, interleukin-2 receptor; ITK, IL-2–inducible kinase; JAK1/2, Janus kinase 1/2; mTOR, mammalian target of rapamycin; HDAC, histone deacetylase; AP-1, activator protein 1; Sirt1, sirtuin 1; Tregs, regulatory T cells; Ab, antibody; Th1, Type 1 T-helper; ROCK2, Rho-associated coiled-coil kinase 2; BLNK, B cell linker; NF-κB, nuclear factor kappa-light-chain-enhancer of activated B cells; NFAT, nuclear factor of activated T cells; ITAMS, immunereceptor tyrosine-based activation motifs; CSF-1R, colony-stimulating factor 1 receptor; BCR, B cell receptor; Btk, Bruton tyrosine kinase; Syk, splenic tyrosine kinase; BAFF, B cell activating factor; BAFF-R, BAFF receptor; ERK, extracellular signal-regulated kinase; CD20, cluster of differentiation 20; CD40L, cluster of differentiation 40 ligand; Ag, antigen; IL-6R, interleukin-6 receptor.

In the pathogenesis of cGvHD, the disease develops as a result of complex molecular networks, from thymus damage to unusual antigen presentation and aberrant T- and B cell interactions (**Figures 1A, B**). This consequently leads to an enhanced Th17 differentiation, macrophage sequestration in tissues, alloantibody formation, and fibrosis, with the latter highly dependent on nuclear factor kappa-light-chain-enhancer of activated B cells (NF-κB) activation and inflammatory cytokines production (7). Usually, the initial phase starts with release of inflammatory cytokines from the host tissues damaged by the pre-transplant conditioning regimens. These cytokines [interleukin-1 (IL-1), tumor necrosis factor alpha (TNF-α), interferon gamma (IFN-γ), platelet-derived growth factor (PDGF)] in combination with antigen presenting cells, stimulate the activation of donor allo-reactive T cells, with expansion of donor helper T cells, cytotoxic T cells and natural killer (NK) cells that cause cytotoxicity against host cells. In addition, both colony-stimulating factor-1 (CSF-1) and granulocyte macrophage-colony stimulating factor (GM-CSF), two key cytokines that modulate the differentiation, proliferation and survival of macrophages, have been implicated in cGvHD (8, 9). The role of GM-CSF activated myeloid cells in mediating GvHD by favoring IL-1β and Reactive Oxygen Species (ROS) production by phagocytes has recently been highlighted (10). This tissue damage is not counterbalanced by the physiologic control exerted by the host thymus and of T regulatory lymphocytes (Tregs) that usually are fundamental for immune tolerance (11). Donor macrophages may also infiltrate organs like the skin and lungs of a HSCT recipient through CSF-1 receptor signaling; and studies have shown that these donor CSF-1R-dependent macrophages may contribute to both sclerodermatous (Scl)-cGvHD and lung cGvHD, otherwise known as bronchiolitis obliterans syndrome (BOS), *via* the expression of Transforming Growth Factor beta (TGF-β) (8). This is important because it is now well established that TGF-β is a fundamental pathogenic cytokine in fibrosis, and elevated levels of this cytokine has been found in cGvHD patients; though the mechanism through which it contributes to the pathogenesis of the disease remains elusive (3). It is, however, clear that in certain organ-specific cGvHDs, such as skin cGvHD, both the TGF-β and PDGF pathways appear to be up-regulated leading to the activation and differentiation of fibroblasts into alpha-smooth muscle actin (α-SMA)-expressing myofibroblasts. These α-SMA-expressing myofibroblasts then proliferate and mediate fibrosis in Scl-cGvHD (12, 13).

Fibroblasts are also fundamental in the pathogenesis of cGvHD involving lacrimal glands, in a different way in respect of Sjogren syndrome. Indeed, fibroblasts act as antigen presenting cells and communicate with various inflammatory cells leading to the invasion of ductal epithelium with its destruction and ductal ectasia of lacrimal glands (14). The involvement of the eyes is so clinically relevant because worsening of cGvHD score in the eyes, joints/fascia, or oral mucosa, when assessed at 6 months, are more likely to predict subsequent treatment failure; with 74% patients free from failure at 36 months when no impairment of symptoms and signs were observed at 6 months *vs* 26% of those that presented a higher eye, mouth or joints involvement and inflammation (15).

The “hyperinflammatory” status that characterizes cGvHD has been known for many years; in 2012, a group from Bethesda elaborated on a simple but effective score for predicting the severity of cGvHD using some parameters typical of autoimmune diseases, such as C reactive protein (CRP), complement (C3 and C4), platelets and albumin levels. When CRP is >0.7 mg/dl, C3 >140 mg/dl, C4 >28 mg/dl, platelets >250 K/μl and albumin <3.6 g/dl were all present, the chances of active cGvHD was 80% *vs* 69% when 1–3 parameters were present and 0% when all variables were lower than the cut off (16).

Moreover, an interesting association between the “inflammatory phenotype” of patients with cGvHD and microbiome composition is emerging. Indeed, gut microbiota of healthy patients has been compared with subjects who underwent allo-HSCT but did not develop cGvHD. The first cohort was enriched with *Bacteroides*, *Lactobacillus*, *Clostridium*, and *Veillonella*, whereas, in the group without cGvHD, two were enriched with *Ruminococcus*, *Blautia*, and *Faecalibacterium*. The latter is a well-known anti-inflammatory commensal bacterium, which was previously reported in patients with intestinal inflammatory diseases including active ulcerative colitis, and its presence correlated with higher amounts of IL-10 (17).

This is the prerequisite for employing anti-inflammatory agents (such as Btk inhibitors, JAK1/2 inhibitors or hydroxychloroquine) in the therapy of cGvHD as described below.

The psychological and economic impact of cGvHD is still enormous, as many complications can emerge from both the disease and its treatment (18). Chronic GvHD impairs the quality of life of the affected patients who require a continued medical follow-up, with higher risk of infection and death. Even the administration of corticosteroids is problematic: in a cohort of 162 patients, it was clearly demonstrated, that cGvHD and steroid treatment significantly prolonged time to recover normal body mass index (BMI) and muscle strength after HSCT (19). Acute GvHD occurs in 40%–50% of patients receiving allo-HSCT, which makes aGvHD a major risk factor for developing cGvHD (20). Other known risk factors include specific diseases, such as chronic myeloid leukemia (CML), age (older patients are more prone to cGvHD), use of mismatched or unrelated donors compared to matched sibling transplants, use of peripheral blood stem cells instead of bone marrow stem cells as a graft source, and sex mismatch between recipients (especially male) and donor (especially female) (21, 22). This review therefore, provides the current knowledge on the emerging therapies for cGvHD, with particular emphasis on TKIs, JAK1/JAK2, and proteasome inhibitors that are now ready for entering into clinical practice.

## Treatment of Chronic Graft-Versus-Host Disease

Standard of care in the treatment of cGvHD depends on the particular organ(s) or site(s) that is/are affected and adopted treatments can be topical or systemic. Nevertheless, about 50%–60% of patients with cGvHD will require a second-line treatment within 2 years, but at the moment there is no consensus on the optimal choice of agents for second or further lines of therapy (23). The National Comprehensive Cancer Network (NCCN) guidelines (https://www.nccn.org/professionals/physician_gls/pdf/hct.pdf) and the European Society for Blood and Marrow Transplantation (EBMT) consensus (24), both edited in 2020, agree in the use of steroids as first-line treatment, and sustain the use of ibrutinib, the only compound approved by the FDA for second- or further line treatment of cGvHD. Nevertheless, both guidelines clearly state that there are still no standard therapies for steroid-resistant (SR) patients, limiting the patients to the listed available drugs (see **Table 1**), and advising clinicians to possibly enroll these patients into clinical trials.

**Table 1:** Possible therapeutic approaches for steroid-resistant/refractory cGvHD patients according to the NCCN and EBMT 2020 guidelines (24).

Drug	Response (%)	Target sites	Adverse events	Notes
Ibrutinib	ORR 69	skin, mouth, GI	bleeding, diarrhea, fatigue, pneumonia, nausea, hematological tox, spasms	approved by FDA advised also by EBMT
Extracorporeal photopheresis (ECP)	ORR 53–61	skin, liver, eyes, mucosa		EBMT advised
Calcineurin inhibitors	ORR 35–46	skin, liver, GI	kidney, hypomagnesaemia, hypertension, tremors	EBMT advised
Mycophenolate mophetyl	ORR 75	skin, mouth	abdominal cramps, infections	EBMT advised
JAK1/2 inhibitors	ORR 43–85	skin, mouth, lung, joints	cytopenias, CMV reactivation	EBMT advised
Low-dose methotrexate	ORR 70	skin, mouth	cytopenias	Expert opinion
Rituximab	ORR 66	skin, mouth, liver, lungs	infusion reactions, infections	EBMT advised
Alemtuzumab	ORR 40–70	skin	infections	Expert opinion
mTOR inhibitors	ORR 63–81	skin, mouth, liver, eyes, GI	thrombotic microangiopathy	EBMT advised
IL-2	ORR 50–60	skin, liver, GI, lung, joints	flu-like syndrome	Expert opinion
TKIs	ORR 36	skin, GI, lung	edema, fatigue, ipophosphatemia	EBMT advised
Etanercept	ORR 62	lung	infections	Expert opinion
Abatacept	ORR 44	mouth, GI, joints, skin, eyes, lung	infections, diarrhea, fatigue	Expert opinion
Hydroxychloroquine	ORR 53	skin, mouth, liver	retinopathy	Expert opinion
Pentostatin	ORR 55	skin, mouth, muscles, GI	renal, fatigue, nausea, infections	EBMT advised

EBMT, European Society for Blood and Marrow Transplantation; NCCN, National Comprehensive Cancer Network; ORR, overall response rate; IL-2, interleukin-2; JAK, Janus associated kinases; GI, gastro-intestinal; TKIs, tyrosine kinase inhibitors; CMV, cytomegalovirus; mTOR, mammalian target of rapamycin.

It also must be noted that cGvHD severely impairs the quality of life. Hence, it is imperative that new treatment options be firstly aimed at improving the quality of life by reducing symptoms, preventing immune-mediated damage and disability, while at the same time avoiding toxicities that are associated to the treatment itself (25, 26). The long-term goal of cGvHD treatment on the other hand, is to institute an immunologic tolerance that will allow the patient to successfully withdraw from immunosuppressive treatments without relapse or clinically significant manifestations of disease activity while preserving the graft-versus-leukemia (GVL) effect (25, 26).

The current available treatment options for cGvHD firstly involve the use of corticosteroids. Due to their lymphopenic and anti-inflammatory properties, and generally based on controlled clinical trials, corticosteroids (such as prednisone) have been the mainstay of first-line treatment of cGvHD for the past three decades (27, 28). These corticosteroids can be administered alone or in combination with other immuno-suppressants such as calcineurin inhibitors, which suppress the immune system by preventing the production of IL-2 by T cells (26–29). This treatment option is sometimes however problematic as it is often inadequate or toxic, and can cause immunosuppression that may lead to an increase in malignancy relapse. Side effects, such as infections, myopathy, cataracts, hyperglycemia, decline in bone mass, and avascular necrosis have all been associated with prolonged corticosteroid use. Combination therapy with immuno-suppressants to reduce these side effects has shown little or no benefit (27, 28). Furthermore, deteriorating signs of cGvHD in previously affected organs along with the development of signs and symptoms of the disease in previously unaffected organs have both also been associated to corticosteroid treatment (26); all of which warrants a second line of treatment in nearly 50%–60% of patients who experience reoccurrence of cGvHD (16). Second-line cGvHD treatment is based on retrospective analyses, and in some cases, single-arm phase II trials (23).

Whatever line of therapy, the treatment of cGvHD has three different goals:

- **to reduce the activated status of B- and T cells**: in this context, Btk inhibitors can reduce the pre-germinal B cells and T follicular helper (Tfh) lymphocytes; JAK1/2 inhibitors can decrease Th1, Th2, and Th17 activity, and anti-B and anti-T cells monoclonal antibodies (mAbs) seem to be also effective. Among the latter drugs, anti-CD20 and anti-CD52 antibodies have already entered into the clinical practice and the anti-CD26 mAb Begelomab seems to be very promising (30).
- **to play an anti-inflammatory effect**, in particular by reducing secretion of IL-6, TNF-α and IL-17. In this category, the most frequently adopted antibodies are Infliximab and Etanercept along with the JAK1 inhibitor Itacitinib.
- **to slow down the development of fibrosis**: in addition to macrophages and pathogenic TGF-β, recent studies have also suggested that fibroblasts isolated from cGvHD patients can promote fibrosis through the upregulation of collagen genes (*COL1α1* and *COL1α2*), a mechanisms which was induced by hyperactive TGF-β signaling (31). In this context, inhibitors of CSF-1, the TGF-β pathway, PDGF, spleen tyrosine kinase (Syk), Rho-associated coiled-coil kinase 2 (ROCK2) and Hedgehog seem promising approaches [see in the following references (3, 32, 33)].

**Table 2** highlights some of the agents that are currently being evaluated in patients with cGvHD. The use of some of these agents to treat cGvHD is based on their current usage in the clinic to treat certain autoimmune diseases, where their tolerability and efficacy profiles are known. In addition, patients with cGvHD often display symptoms resembling autoimmune diseases, making these agents appealing for cGvHD treatment. The mechanism of action of these agents on both B and T cells in cGvHD is illustrated in **Figure 1C**.

**Table 2:** Selected ongoing studies evaluating novel drug agents in patients with cGvHD.

Agent	Agent type	Target	ClinicalTrial.gov identifier	Clinical trials (status)	Clinical trials (status)
Imatinib	TKI	BCR-ABL, inhibits B cell signaling	NCT01309997	II	Imatinib treating cutaneous sclerosis in patients with cGvHD
Nilotinib	TKI	BCR-ABL, inhibits B cell signaling	NCT01810718	I/II	Nilotinib for treating patients with steroid-refractory cGvHD
Ibrutinib	TKI	Btk, Syk, Itk	NCT03790332	I/II	Dose/safety study of ibrutinib in pediatric patients with cGvHD
Fostamatinib	TKI	Syk, inhibits B cell signaling	NCT02611063	I	Fostamatinib - preventing and treating cGvHD after allogeneic stem cell transplant
Entospletinib (ENTO)	TKI	Syk, inhibits B cell signaling	NCT02701634	II	Entospletinib + systemic corticosteroids as first-line therapy for cGvHD
Itacitinib	TKI	JAK1	NCT03584516	III	Itacitinib + corticosteroids as initial treatment for cGvHD
Ruxolitinib	TKI	JAK1/2	NCT03395340	II	Topical ruxolitinib for cGvHD
Ruxolitinib	TKI	JAK1/2	NCT03616184	II	Ruxolitinib for sclerotic cGvHD after failure of systemic steroid therapy
Baricitinib	TKI	JAK1/2	NCT02759731	I/II	JAK1/2 Inhibition in cGvHD
Vismodegib	TKI	Hedgehog, inhibits SMO	NCT02337517	N/A	Vismodegib for treating patients with steroid-refractory cGvHD
Sonidegib (LDE225)	TKI	Hedgehog, inhibits SMO	NCT02086513	I	Sonidegib for treating SR-cGvHD after allo-HSCT
Ixazomib	Proteasome inhibitor	26S Proteasome	NCT03225417	I/II	Ixazomib + rapamycin and tacrolimus in the prophylaxis of cGvHD
Bortezomib (Velcade)	Proteasome inhibitor	Suppresses T cell, proliferation and growth and inhibits B cell activation *via* NF-κB inhibition	NCT00815919	II	Bortezomib + Prednisone as initial treatment for cGvHD
Carfilzomib	Proteasome inhibitor	Suppresses T cell, and B cell development, activation and survival	NCT02491359	II	Carfilzomib therapy for cGvHD
KD025	ROCK2 inhibitor	ROCK2, pSTAT3, IL-17 and IL-21 inhibition	NCT03640481	II	ROCK2 inhibition in cGvHD after at least two prior lines of systemic therapy
KD025	ROCK2 inhibitor	ROCK2, pSTAT3, IL-17 and IL-21 inhibition	NCT02841995	II	To evaluate, safety, tolerability and activity of KD025 in patients with cGvHD
Panobinostat (LBH589)	Deacetylase inhibitor	Inhibits HDAC	NCT01028313	II	LBH589 as second-line therapy for patients with cGvHD
Rituximab	Monoclonal antibody	Anti-CD20, depletes B cells	NCT01161628	II	Safety/efficacy of rituximab as primary treatment for extensive cGvHD
Ofatumumab	Monoclonal antibody	Anti-CD20, depletes B cells	NCT01680965	I/II	Safety/side effects of ofatumumab in cGvHD
Axatilimab (SNDX-6352)	Monoclonal antibody	Blocks CSF-1R	NCT03604692	I	Investigate SNDX-6352 in subjects with active cGvHD
Abatacept	Fusion protein	Inhibits the CD28 signaling pathway of T cell.	NCT01954979	I	Abatacept for treating patients with steroid-refractory cGvHD
Alefacept	Fusion protein	Inhibits T cell activation and proliferation	NCT01226420	II	Alefacept for steroid-refractory cGvHD
IL-2	Cytokine	Tregs development and expansion	NCT01366092	II	Low-dose IL-2 for steroid-refractory cGvHD
ECP + Aldesleukin (Proleukin)	ECP + Cytokine	Expands Tregs, NK-cells	NCT03007238	II	ECP + low dose IL-2 (interleukin 2) (aldesleukin)
AMG 592	IL-2 mutein	Expands Tregs, NK-cells	NCT03422627	I	Safety/efficacy of AMG 592 in patients with steroid-refractory in cGvHD
Ibrutinib + Rituximab	TKI + Monoclonal antibody	Btk, Syk, Itk, Anti-CD20, depletes B cells	NCT03689894	I	Combining ibrutinib and rituximab for the treatment of cGvHD
PredEver	Immunosuppressant + mTOR inhibitor	Glucocorticoid receptors, inhibit IL-2 mediated activation of B and T cells	NCT01862965	II	Prednisone + Everolimus for treating patients with moderate to severe cGvHD

TKI, tyrosine kinase inhibitor; aGvHD, acute graft versus host disease; cGvHD, chronic GvHD; Btk, Bruton tyrosine kinase; Itk, Interleukin-2-inducible kinase; Syk, spleen tyrosine kinase; JAK, Janus associated kinases; BCR-ABL1, breakpoint cluster region-Abelson; CSF-1R, colony-stimulating factor 1 receptor; ROCK2, Rho-associated coiled-coil kinase 2; IL-2, interleukin-2; pSTAT3, phosphorylated-signal transducer and activator of transcription 3; mTOR, mammalian target of rapamycin; CD20, cluster of differentiation 20; NF-κB, nuclear factor kappa-light-chain-enhancer of activated B cells; NK, natural killer; Tregs, regulatory T cells; HDAC, histone deacetylase; ECP, extracorporeal photopheresis; HSCT, hematopoietic stem cell transplantation; SMO, smoothened; N/A, not applicable.

## Rationale for Targeting B Cells

It is now quite clear that in addition to alloreactive T cells, allo- and auto-reactive B cells along with several cytokines, play a fundamental role in the development of cGvHD (1, 3, 34–39). Undoubtedly, a central event in the pathogenesis of cGvHD is the recognition of antigen (Ag) *via* the B cell receptor (BCR). A major difference to the physiological recognition of antigen by the BCR has been demonstrated in the murine model, where B cells have a BCR-hyper-responsiveness in mice developing cGvHD (36–39). When pathogenic B cells are activated in cGvHD, they expand in a process that is guided by soluble factors, including IL-4, IL-17, IL-21, and B cell activating factor (BAFF), which is connected to the formation of germinal centers (GCs) (37, 40, 41). Donor Tfh cells cooperate with GC B cells that undergo somatic hypermutation by amassing B cells capable of producing antibodies to antigens, thereby, activating the BCR and thus, supporting cGvHD [as reviewed in (36)]. The increased number of circulating Tfh cells may thus serve as a surrogate marker of the presence of these Tfh cells in GCs: in a cohort of 70 patients, the number of circulating Tfh cells increased in patients with cGvHD, especially in those with higher severity of disease, where the expansion of Tfh was highly correlated with the generation of IgG1 antibodies (36).

Furthermore, clinical studies have demonstrated a striking increase of BAFF in the plasma of patients with cGvHD, and also of the BCR intercellular signaling molecules, such as Syk and B cell linker (BLNK) (42), which are relevant for cGvHD development. In preclinical studies, mice lacking Btk failed to develop cGvHD (39). It must be noted that when Syk is phosphorylated upon BCR engagement, it sustains B cell survival and proliferation, and promotes monocyte migration and fibrosis in cGvHD murine models (43). Moreover, after linking to its receptor, BAFF also activates extracellular regulated kinase (ERK), Protein kinase B (Akt), and NF-κB; hence, proteasome inhibitors, such as bortezomib and ixazomib, which are able to block the NF-κB signaling, can be good candidates for the treatment for cGvHD (6).

## Rationale for Targeting T Cells

Chronic GvHD, as mentioned earlier, is an immunologic attack of host organs or tissues by donor T cells and B cells following HSCT. Donor T-helper (Th) cells are crucial in GvHD initiation, owing to their ability to differentiate into Th1 (secreting IL-2 and IFN-γ), Th2 (secreting IL-4, IL-5, IL-10 and IL-13), Th17 (secreting IL-17), and Tfh cells, which facilitate organ-specific GvHD (44). Previously published studies have shown that cGvHD results from the selective activation of allo-reactive donor CD4^+^ T cells that act as helpers to host B cells, thereby triggering B cell activation and auto-antibody production (45). In addition, CD4^+^ Foxp3^+^ regulatory T cells are important mediators of immune tolerance, and their impairment has been associated with cGvHD (46).

Some of the new therapeutic approaches for cGvHD are therefore aimed at targeting T cell responses or T cell signaling pathways. For example, the critical homeostatic cytokine ***IL-2***, at low doses, can be used to target Treg development and expansion as shown in a phase I clinical trial, where 1.5 × 10^6^ IU/m^2^/day of IL-2 was able to selectively target and enhance Tregs, with improvement of patients with cGvHD (47). ***Aldesleukin***, a recombinant human IL-2, in combination with extracorporeal photopheresis (ECP) to treat SR-cGvHD, is also in a phase II clinical trial. Furthermore, the safety and tolerability of ***AMG 592***, an IL-2 mutein, is been evaluated in a phase I/II clinical trial in subjects with SR-cGvHD (ClinicalTrials.gov identifier: NCT03007238, NCT03422627).

At the ASH meeting held in 2018, it was reported that ***artesunate*** (a drug used for treating malaria) improved the survival of mice with cGvHD by increasing the number of Tregs and decreasing that of Th17 both in peripheral blood (PB) and spleen, further sustaining the role of T cells in mediating cGvHD (48).

Other agents targeting T cells include abatacept and alefacept (both of which target and inhibits the activation and proliferation of T cells), the ROCK2 inhibitor KD025 (49), and the proteasome inhibitors ixazomib (50), bortezomib (51), carfilzomib (52), and ibrutinib (53). These agents are discussed in a more detail later in this review.

## The Role of Syk, Btk, and Itk in the Development of cGvHD

Syk, Btk and IL-2-inducible T cell kinase (Itk) are cytoplasmic TKs that are fundamental for the development of B- and T cells, playing multiple functions in the immune system. In general, Btk and Syk are crucial for the phosphorylation and activation of downstream effectors in BCR signaling, while Itk is crucial for the activation of downstream effectors in TCR signaling. Moreover, Syk is involved in immune receptor signaling, such as BCR signaling that promotes B cell proliferation and survival, but also in control of cell migration, cellular adhesion, innate immune recognition, platelet activation and vascular development, while Btk is responsible for B cell development, differentiation and signaling (54). Moreover, Btk is central in control of the Toll-like receptor (TLR)-induced IL-10 expression by B cells, and is an active part of the NLRP3 inflammasome, a multimeric protein complex that triggers the release of proinflammatory cytokines, such as IL-1β and IL-18, in many inflammatory conditions, including Alzheimer’s disease, diabetes, and infections (55), and lack of Btk can lead to a compromised BCR-induced proliferation and survival. Finally, Itk is responsible for T cell development, differentiation and signaling (56).

Recent studies demonstrated that Btk, Itk, and Syk, may all be required for the development of cGvHD (39, 43, 57). It is worth noting that upon engagement of the BCR and TCR with a signaling molecule, SRC family kinases, including Lck/Yes novel tyrosine kinase (Lyn) and lymphocyte-specific protein tyrosine kinase (Lck), are activated leading to the subsequent activation of Syk, Btk and Itk. These activated molecules then activate some transcription factors such as NF-κB in B cells and nuclear factor of activated T cells (NF-AT) in T cells, subsequently controlling cell survival, proliferation, and production of pro-inflammatory cytokines (54, 58) (see **Figure 1B**). This is important in the pathophysiology of cGvHD as Btk activation can drive the production of self-reactive antibody complexes which may be deposited within healthy tissues and blood vessels and hence, promote cGvHD development (**Figure 1A**). Furthermore, pro-inflammatory cytokines produced as a result of Syk, Btk, or Itk signaling can have a direct effect on cGvHD target tissues (58, 59).

## Emerging Therapies for Chronic Graft-Versus-Host Disease

Several agents are currently under investigation for the treatment of cGvHD, which act by either targeting B cells or T cells, or both, and/or macrophages. These agents include TKIs, anti-CD20 monoclonal antibodies and Syk inhibitors, which target B cells, proteasome inhibitors, which target T cells, and JAK1/2 and Btk inhibitors, which target both B and T cells (24). Some of these agents are discussed below according to their known mechanisms of action.

### Inhibition of Tyrosine Kinases

**Figure 2:**
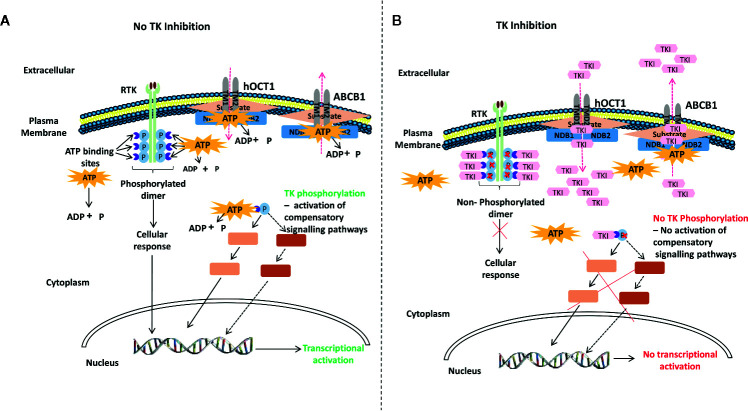
A generalized proposed mechanism of action of tyrosine kinase inhibitors (TKIs). **(A)** auto-phosphorylation of receptor TK (RTK) (light green) upon ligand (brown) binding activates TKs (purple) downstream leading to transcriptional activation. RTK functions by binding ATP and transferring phosphate from ATP to tyrosine residues on various substrates, which leads to their phosphorylation and subsequent cellular response; such as the excess proliferation of cells. Intracellular non-RTKs can also bind ATP and transfer phosphate from ATP to tyrosine residues on various substrates, which can also lead to their phosphorylation and subsequent cellular response. The plasma membrane also has influx and efflux transporters that are ATP-gated (left hand panel). **(B)** In the absence of TK activity, substrates necessary for RTK function cannot be phosphorylated and consequent cellular events are abrogated. Hence, TKIs function by binding to ATP-binding catalytic sites of the TK domains on RTKs or on non-RTKs, thereby, preventing TK phosphorylation and inhibiting down-stream intracellular signaling and transcription of genes such as pro-inflammatory cytokines. TKIs can also bind and occupy ATP- or substrate-binding sites on transmembrane transporters such as the hOTC1 influx protein and the ABC efflux pumps (64–66), thereby, affecting drug and cellular responses (right hand panel). RTK, receptor tyrosine kinase; TK, tyrosine kinase; hOCT1, human organic cation transport member 1; ABC, ATP-binding cassette; ABCB1, ABC sub-family B member 1 (P-glycoprotein 1); ATP, adenosine triphosphate; ADP, adenosine diphosphate; p, phosphate; NDB1, nucleotide binding domain 1; NDB2, nucleotide binding domain 2; TMD1, transmembrane domain 1; TMD2, transmembrane domain 2; TKI, tyrosine kinase inhibitor.

Tyrosine kinases (TKs) have been implicated in several cellular processes including differentiation, proliferation, anti-apoptosis, and B and T cell signaling (28, 53, 60–63). Consequently, the tyrosine kinase inhibitors (TKIs), by blocking B and T cell activation, could be appealing for the treatment of cGvHD. TKIs function by binding to ATP-binding catalytic sites of the tyrosine kinase domains on receptor (RTKs) and on non-receptor TKs. In the absence of TK activity, substrates necessary for RTK function cannot be phosphorylated and consequent cellular events are abrogated. Hence, TKIs can cause a blockade of downstream intracellular signaling and transcription of genes, including some pro-inflammatory cytokines (**Figure 2**).

The efficacy profiles along with the mechanism of action of most of these TKIs are also known, as they have long been used to treat hematological diseases, such as acute leukemia (67), chronic lymphocytic (CLL) (68). In CML, for example, the rearrangement of *BCR* and *ABL1* genes gives origin to a fusion gene where the control of the ABL1 kinase function is lost and so the new protein is constitutively active, with the consequent excess of proliferation of myeloid precursors and an abnormal interaction of the leukemic stem cells with the actin and the bone marrow stroma (69) (**Figure 2**, left hand panel).

TKIs can also bind and occupy ATP- or substrate-binding sites on transmembrane transporters, such as the ATP-binding cassette (ABC) efflux pumps and the human organic cation transport member 1 (hOTC1) influx protein (64–66) thereby affecting drug and cellular responses (**Figure 2**, right hand panel). In CML, TKIs such as imatinib and dasatinib, have been shown to not only target and inhibit BCR-ABL1, but also c-KIT, PDGF receptor (PDGFR), and Src family kinases, which results in the abrogation of the transcription of pro-inflammatory cytokines such as IL-1, IL-6, and TNF-α, and prevents the proliferation of myeloid cells (70).

In cGvHD, TKIs could be used to inhibit growth factor receptor pathways such as those of PDGFR, epidermal growth factor receptor (EGFR) and TGF-β pathways. Indeed, ABL1 is located downstream of TGF-β, and in preclinical models of systemic sclerosis, a disease which may resemble Scl-cGvHD, the production of proteins of the extra-cellular matrix by TGF-β was significantly decreased in ABL1-deficient cells (71). On the other hand, it has been clearly demonstrated that many TKs, such as PDGFR, vascular endothelial growth factor receptor (VEGFR) and EGFR are significantly increased during liver fibrosis development, transforming the hepatic stellate cells from resting to active (63, 72). In addition, other signaling pathways, such as MEK/ERK and phosphatidylinositol-3 kinase (PI3K)/Akt seem to be activated by TKs during hepatic stellate cells activation; this is the basis for employing TKIs in diseases such as liver fibrosis (72) **Table 3**.

**Table 3:** Translation of selected tyrosine kinase inhibition strategies from animal models of cGvHD into clinical trials.

Tyrosine kinase inhibitor	Main conclusion from the preclinical model of GvHD (year)	Reference	Main conclusion from the clinical trials (year)	Reference
Ibrutinib	Inhibition of Btk and Itk with ibrutinib Is Effective in the Prevention and treatment of cGvHD in Mice.	(39, 53, 73)	Both Btk and Itk may be required for the development of cGvHD and inhibition of these signaling molecules by ibrutinib may prevent or even treat cGvHD	(62)
Fostamatinib	Fostamatinib is effective in treating established cGvHD in mice	(38, 43)	Ongoing Fostamatinib—preventing and treating cGvHD after allogeneic stem cell transplant	ClinicalTrial.gov identifier NCT02611063 (43)
Entospletinib (ENTO)	Inhibition of Syk with entospletinib prevents ocular and skin GVHD in mice	(74)	Ongoing Entospletinib—preventing and treating cGvHD after allogeneic stem cell transplant	ClinicalTrial.gov identifier NCT02701634
Ruxolitinib	Ruxolitinib improves the severity cGvHD significantly	(75)	Ruxolitinib may prevent or even treat SR-cGvHD	(76)
Imatinib	Impact Unclear. In one study, imatinib had limited impact on Scl-cGvHD, while in another, it prevented it	(77, 78)	Imatinib may prevent or even treat SR-cGvHD	(79, 80, 81)
Nilotinib	Nilotinib prevents and treats Scl-cGvHD	(95, 96, 78)	Nilotinib may prevent or even treat patients with SR- or steroid-dependent cGvHD	ClinicalTrial.gov identifier: NCT01810718, NCT01155817 (84)
Ixazomib	Ixazomib may prevent GVHD Unknown for cGvHD	(85)	Combination of ixazomib and cyclosporine A may be able to prevent cGvHD	(50)
Baricitinib	Baricitinib prevents and/or reverses established GvHD. Unknown for cGvHD	(86)	Baricitinib may prevent or even treat cGvHD	ClinicalTrial.gov identifier NCT02759731
Vismodegib	Unknown	N/A	Vismodegib may treat SR-cGvHD	ClinicalTrials.gov identifier: NCT02337517 (87)
Sonidegib (LDE225)	Sonidegib prevents and treats Scl-cGvHD	(88)	Sonidegib may treat SR-cGvHD	ClinicalTrials.gov identifier: NCT02086513 (89)

aGvHD, acute graft versus host disease; cGvHD, chronic GvHD; TKI, tyrosine kinase inhibitor; Btk, Bruton tyrosine kinase; TKI, tyrosine kinase inhibitor; Itk, Interleukin-2-inducible kinase; Syk, spleen tyrosine kinase; Scl-cGvHD, Sclerodermatous cGvHD; SR-cGvHD, steroid-refractory cGvHD; N/A, not applicable.

***Imatinib*** is a first generation TKI approved for the treatment of CML and Philadelphia-positive acute lymphoblastic leukemia (ALL) and for myelodysplastic/myeloproliferative diseases carrying the PDGFR rearrangement. In addition to the BCR-ABL1 fusion protein, imatinib can also target and inhibit PDGFR and TGF-β pathways both of which play important roles in the fibrosing process occurring in Scl-cGvHD. Hence, targeting these growth factors could represent an important therapeutic strategy for treatment of cGvHD (90, 91).

In a murine model of bleomycin-induced fibrosis, imatinib prevented the differentiation of fibroblasts into myofibroblasts and reduced the synthesis and accumulation of proteins of the extra-cellular matrix in the skin, significantly ameliorating the mice phenotype (92, 93). Besides, imatinib was also effective against pulmonary, renal and liver fibrosis. In the same model, imatinib significantly prevented pulmonary fibrosis by inhibiting proliferation of mesenchymal cells (93).

In one preclinical study, Belle *et al*. used a murine model of Scl-cGvHD to assess the therapeutic impact of imatinib; published data indicated that imatinib is able to reduce the proliferation of both total T cells and T-regs in the spleen. Nevertheless, despite the inhibition of PDGFR, there was no significant clinical impact of imatinib on murine Scl-cGvHD (77). The impact of imatinib on liver fibrosis is more debated: indeed, it significantly decreased the expression of some fibrosis markers, such as α-SMA, Pcol1A1, and Hsp47 (94), but reduced only the early liver fibrogenesis without preventing the long-term fibrosis progression (95). Moreover, in a small series of CML patients, our group recently showed that imatinib is able to reduce the expression of several genes that usually are highly expressed in autoimmune diseases. Genes such as *ANX4A*, which is highly expressed in the Sjogren’s syndrome, *CASP10*, highly expressed in Crohn’s disease, *CEACAM8* and *CTSG*, overexpressed in arthritis, *ITGAM*, elevated in psoriasis, and *PGLYRP1*, whose high levels have been documented in chronic gingivitis, were all reduced by imatinib; thus, supporting the “anti-inflammatory” power of this drug (96).

In 2002, it was reported for the first time that in a series of 23 CML patients with bone marrow fibrosis, treatment with imatinib for 3 months induced a marked regression of bone marrow fibrosis (97). Two years later, another group demonstrated that imatinib reduced fibrosis by at least 2 grades in 61% and by at least 1 grade in 85% of cases, independently on the cytogenetic response (98). Similar successes were reported in patients with treatment-resistant systemic sclerosis, where skin fibrosis generally was more responsive than lung fibrosis. In this context, the authors showed that imatinib decreased the phosphorylation both of PDFGR and of c-ABL1 in the skin of systemic sclerosis (SSc) patients (99). This finding is particularly interesting since SSc may resemble Scl-cGvHD, which paves the way for a similar imatinib treatment approach in Scl-cGvHD patients.

On this basis, in 2009, Olivieri *et al*. reported the efficacy of imatinib at 200 mg/day in a series of 19 patients with refractory cGvHD, where the organs mostly involved were skin, lungs and bowel. After 6 months of treatment, half of the patients showed a significant improvement of cGvHD signs, especially in skin fibrosis (79). The same conclusions were drawn by Magro et al. who reported that imatinib 400 mg/day was able to reduce skin sclerosis within 2 months of treatment. They investigated the safety and efficacy of imatinib in 14 patients with Scl-GVHD: with a median follow-up of 11.6 months, ORR was 50%, including 28% of CR and 71% of PR and a significant reduction of steroid dose. Imatinib was well tolerated in most patients but in 29% of them, adverse events (muscle cramps, nausea diarrhea, and fatigue) led to treatment discontinuation (100). In addition, there is some evidence to suggest that imatinib can improve the severity of cGvHD and at the same time allow for the withdrawal of systemic corticosteroids without further deteriorating the disease symptoms (79, 80, 99, 101, 102).

A possible explication of these different results could be probably found in some pharmacogenetic aspects. Imatinib is substrate of transmembrane influx and efflux pumps, as clearly showed in CML where the combination of polymorphisms of the human organic cation transporter type 1 (hOCT1) influx pump and of one of the most important efflux pumps (ATP-binding cassette sub-family B member 1; ABCB1) seem to influence either the plasma level or the drug intra-cellular concentrations, with a clear impact on toxicity and efficacy (103). Analogously, the impact of transporters in the intake of imatinib by dermal fibroblast has been recently assessed by a German group: OCT1, OCT2, organic cation transporter novel type 1 (OCTN1), OCTN2, and Multidrug and toxin extrusion 1 (MATE1) were expressed in dermal fibroblasts and the expression and activity of MATE1 were deemed responsible for imatinib uptake. On the other hand, MATE1 expression seemed to be correlated to cytokines, such as PDGF subunit B (PDGFB), and to the NOTCH signaling pathway. Indeed, in dermal fibroblasts, the NOTCH pathway is highly activated and blockade of this signaling network normalized the transporter function of MATE1 and increased the imatinib efficacy in fibroblasts from patients with SSc (104).

***Nilotinib*** is a second-generation FDA approved TKI that could also be used as first-line treatment of CML. Like imatinib, it targets BCR-ABL1 and PDGFR with a higher affinity and lower IC_50_ compared with imatinib, so offering higher rates of early and deep molecular responses—conditions necessary also for a further interruption of treatment (105–108). In preclinical cGvHD models, nilotinib has been able to prevent Scl-cGvHD by targeting and inhibiting the aberrant activation of the pro-fibrotic c-ABL1 kinase and PDGFR (82, 83).

In another murine model of liver fibrosis, nilotinib decreased TNF-α, TGF-β, receptor for advance glycation endproducts (RAGE), and high-mobility group box 1 protein (HMGB1) mRNA expression, decreased nitric oxide levels and increased glutathione peroxidase activity, with significant reduction of fibrosis (83).

The anti-inflammatory and immunomodulatory effects of nilotinib on cGvHD were further highlighted when cultured CD3-positive peripheral blood mononuclear cells from healthy donors were exposed to varying concentrations of this TKI. Nilotinib suppressed the production of pro-inflammatory cytokines, including IL-2, IL-10, IL-17, IFN-γ and TNF-α; nevertheless, it had little or no effect on the frequency of Tregs, B and NK cells (109).

Elevated levels of TGF-β as mentioned earlier, has been found in cGvHD patients though the mechanism through which it contributes to the pathogenesis of the disease remains elusive. In their study, Busilacchi *et al*. demonstrated that nilotinib represses the growth of fibroblast isolated from cGvHD patients and causes earlier senescence in these cultured cells compared to normal fibroblasts. More importantly, nilotinib treatment led to the downregulation of both *COL1α1* and *COL1α2*, along with TGF-β inhibition, which was associated with reduction of intracellular phospho-Smad2. Hence, suggesting that TGF-β inhibition at intracellular and systemic level denotes a crucial anti-fibrotic apparatus of nilotinib in a clinical setting (31).

Recently, Olivieri *et al*, conducted a phase I/II study in which they evaluated the safety and maximum tolerated dose (MTD) of nilotinib in 22 SR-cGvHD patients with multiorgan involvement. MTD could not be reached after dose increase of up to 600 mg/day, and according to the 2005 and 2014 NIH and GITMO [an exploratory approach, combining objective involvement (OI) without failure] response criteria, ORR at 6 months were 27.8%, 22.2%, and 55.6%, respectively. Overall survival (OS) at 48 months was 75%, whereas, the failure free survival (FFS), according to the NIH and GITMO criteria was 30% and 25%, respectively. Adverse events that were attributed to nilotinib treatment included itching, headache, nausea, cramps, mild anemia, and asthenia. Nonetheless, it was concluded that the safety profile of nilotinib and long-term outcome makes this TKI an attractive option in SR-cGvHD (110).

There are currently a number of ongoing phase I/II studies that are aimed at evaluating the safety and efficacy of nilotinib in patients with steroid-refractory or steroid-dependent cGvHD (ClinicalTrials.gov identifiers: NCT01810718, NCT01155817).

### Btk and Itk Inhibition

***Ibrutinib*** is a first-generation TKI which in humans, targets and impedes the activation of B and T cells signaling by binding to and inhibiting Btk and Itk, respectively (31, 65). In a phase II trial, ibrutinib offered to SR cases resulted in 67% of rapid multiorgan responses (62). No major hemorrhages were reported and atrial fibrillation was reported only in 2% of cases. Overall, treatment discontinuation rate by 2 years of treatment occurred in one-third of cases. Ibrutinib is approved by the FDA for the treatment of SR-cGvHD, and recently, this drug showed a significant improvement of quality of life in a series of 42 patients affected by cGvHD (111).

### Syk Inhibition

***Fostamatinib***, like ibrutinib, is a small molecule first-generation TKI, but unlike ibrutinib, fostamatinib is able to ameliorate the severity of cGvHD by targeting Syk and blocking its down-stream signaling. It also prevents the production of chemokines and pro-inflammatory cytokines that are involved in the activation of CD4^+^ T cells and their subsequent differentiation into a Th1 or Th17 phenotype (36, 38, 43). Using mouse models of cGvHD with multiorgan system, Flynn *et al*. identified Syk-mediated BCR signaling in murine allogeneic B cells as a facilitator of cGvHD and they further validated targeting and inhibiting Syk with fostamatinib as an effective approach for the treatment of cGvHD (43). In a similar study, using a murine model of Scl-cGvHD, Huu *et al*. observed Syk activation and phosphorylation in B and T cells, as well as macrophages after allo-HSCT, and Syk inhibition using R788 (fostamatinib sodium) significantly reduced the severity and fibrosis of Scl-cGvHD. They further showed that R788 was not only effective at inhibiting the production of IL-6 by macrophages, but also production of IL-13, IL-17A, and IFN-γ by CD4^+^ T cells (38).

There is currently no reported clinical study highlighting the effects of fostamatinib in cGvHD. However, based on results from *in vitro* and *in vivo* preclinical data in cGvHD (38, 75), fostamatinib is currently being evaluated for the treatment and prevention of cGvHD in a phase I clinical trial. Enrolled patients will receive fostamatinib 100 mg qd, 150 mg qd, or 100 mg bid from day +90 days after transplant and up to 1 year (ClinicalTrials.gov identifier: NCT02611063). Results of this trial have not yet been presented.

### Blockade of JAK1/2 Signaling

Preclinical and clinical data suggest that JAK1/2 signaling may play an important role in the pathogenesis of B- and T cell-mediated GvHD (75, 113). This notion is supported by the requirement of a constant activation of cytokine receptors that are necessary for the production of a number of key cytokines which are implicated in the pathogenesis of GvHD by JAK1/2.

***Ruxolitinib***, a JAK1/2-inhibitor, has been used to suppress T cell activation *via* inhibition of cytokine receptor-mediated signaling. Inhibition of this pathway by ruxolitinib improves the outcome of subjects affected by cGvHD not only in murine models, but also in patients (75). JAK1/2 inhibition by ruxolitinib and the resulting attenuation of several murine cGvHD features, is possibly in part through the down-regulation of IFN-γ signaling (75). This drug has been reported to be effective also against the BOs, characterized by fixed airway obstruction and bronchiolar inflammation. In a series of five pediatric patients, two had a partial response (PR), and one an improvement in FEV1 rapidly after starting ruxolitinib (114). In addition, a recent meta-analysis, which included eight different studies that was focused on cGvHD, reported that ruxolitinib offered 62% of overall responses, with 27% of complete and 45% of partial resolution of GvHD signs and symptoms (115).

***Itacitinib*** is a powerful anti JAK1 inhibitor that is effective in treating connective tissue diseases (116). In a phase-1 study on aGvHD, when administered at 200 or 300 mg, it induced 78.6% and 66.7% of overall response rate, respectively, with 70.6% of successes in steroid-refractory patients (117). On this basis, two studies trying itacitinib in cGvHD recently started the accrual (ClinicalTrials.gov identifier: NCT04200365, NCT03584516).

### Proteasome Inhibitors

***Bortezomib*** is a proteasome inhibitor, which in a phase II trial, administered at 1.3 mg/m^2^ i.v. on days 1, 8, 15, and 22 of each 35-day cycle for 3 cycles, was used in combination with prednisone for initial therapy of cGvHD. This combination was well tolerated, with limited occurrence of sensory peripheral neuropathy. The ORR was 80%, with only 10% of CRs, especially in skin (73%), gastrointestinal tract (75%), and liver (53%). One-third of cases showed a significant improvement of symptoms referred to joints, muscles, or fascia (118). More recently, it has been reported that bortezomib is well tolerated and effective in pulmonary cGvHD: in 17 patients, with a cGvHD lasting from more than 3 years, bortezomib induced a significant reduction of forced expiratory volume (FEV1) decline (from -1.06%/month before to -0.25%/month after treatment) (119).

***Ixazomib*.** Interesting data are coming from the murine model where ixazomib, a second-generation proteasome inhibitor, has been used: it suppressed naïve human dendritic cells (DC) maturation, reducing their pro-inflammatory cytokine production, with a final improved survival of mice (85). Ixazomib was also able to induce apoptosis of activated T lymphocytes, reduce effector CD4+ T cells in bone marrow and spleen, increase Tregs in lymph nodes, Peyer patches and thymus, so suggesting the possibility of using this drug, usually employed in relapsed multiple myeloma (120), for treating cGvHD (50). When administered at 4 mg on days 1, 8, and 15 of a 28-day cycle for up to six total cycles, ixazomib showed 34% of ORR at 6 months; overall survival (OS) at 6 and 12 months was 92% and 90%, respectively. Interestingly, this therapy was well tolerated, because no subjects discontinued the treatment by 12 months (121). Moreover, after ixazomib exposure, serum BAFF levels decreased, so supporting a possible immune modulatory role for this new drug (122).

### Monoclonal Antibodies and Fusion Proteins

Like the TKIs, the efficacy profiles along with the mechanism of action of most of these mAbs and fusion proteins are known, as most of them are currently employed in the clinic to treat autoimmune diseases, such as rheumatoid arthritis (105). These compounds have been used to target various immune cells including B cells, T cells and macrophages in cGvHD settings. Rituximab, obinutuzumab, and ofatumumab are all anti-CD20 mAbs that have been shown to deplete B cells and thereby prevent B-cell clonal expansion *in vivo* cGvHD settings (106). The anti-CD52 mAb Alemtuzumab, which targets and deplete B cells, T cells, dendritic cells (DCs), and natural killer (NK) cells, is also promising for the treatment of cGvHD (107). In addition, a number of studies have reported the use of fusion proteins to treat cGvHD (108, 123). The use of some of these mAbs, antibody-drug conjugates, and fusion proteins for the treatment of cGvHD are discussed below.

***Rituximab*** is a chimeric mAb that targets CD20, a cell-surface marker specifically found on pre-B and mature B-lymphocytes. Binding of rituximab to CD20 results in destruction of the lymphocytes by several mechanisms, including antibody-dependent cell-mediated cytotoxicity, complement-dependent cytotoxicity and direct apoptosis (124, 125). It is usually employed at 375 mg/m^2^ weekly for four doses; it represents the mAb that is most frequently employed in cGvHD treatment (126–128), including Scl-cGvHD (129) in addition to other types of cGvHD (37, 130, 131). Recently, it has been reported that rituximab offered 41% of responses at 1 year, 69% at 2 years, and 77% at 3 years. Interestingly, 67% of responsive subjects tapered steroids. The probability of being alive and free from cGvHD was 36%, 55%, and 57% at 1, 2, and 3 years, respectively (132).

Finally, combining B cell depletion with rituximab (375 mg/m^2^, once per week, for 4 weeks) followed by TKI inhibition with nilotinib (200 mg, twice daily, for 6 months) have shown a more profound and long-lasting response (8% CR, 63% PR) with a higher survival rate (96.6% in 1 year) in patients with Scl-cGvHD (129).

***Ofatumumab*** is another fully human anti-CD20 mAb, which, like Rituximab, targets, and depletes B lymphocytes (133). Administration of ofatumumab alone or in combination with glucocorticoids has demonstrated clinical improvement of cGvHD symptoms in different studies (134). In a small series, Ofatumumab was administered at 1000 mg on days 1 and 14 without dose-limiting toxicity. Fatigue, transaminitis, and infusional reactions were observed, but there were no cases of hepatitis B reactivation or progressive multifocal leukoencephalopathy. At 6 months, the ORR was 72%, with 36% of complete responders (CRs) and 90% of patients who reduced the steroid dose (135).

***Obinutuzumab*** is a novel powerful humanized anti-CD20 mAb. A randomized study with this anti-CD20 antibody, infused at 3, 6, 9, and 12 months from transplantation, is now opened to enrollment (ClinicalTrials.gov identifier: NCT02867384).

*Begelomab* is a murine IgG2B mAb against CD26 that usually favors T cell migration. In a series of 28 patients, begelomab was administered at 2–4.5 mg/day for 5 days, with possible 6 additional doses in 3 weeks, and this cohort was compared with 82 matched controls who received anti-thymocyte globulin, ECP, mycophenolate, sirolimus, etanercept, high dose cyclophosphamide. Begelomab rendered a more effective result than conventional treatments, with an ORR of 75% *vs* 41%, with particular improvement in the gut (ORR 82% *vs* 34%). This translated into an advantage in terms of outcome, with 1-year OS of 50% *vs* 31% of the controls (136). In another recently published study, begelomab was shown to induce responses in over 60% of SR-aGvHD patients, including those with severe gut and liver GvHD, having failed one or more lines of treatment. Begelomab was administered to 69 patients with SR-aGvHD; of which, 8, 33, and 28 had severity of GvHD grades II, III, and IV, respectively. Notably, 28 of the 69 patients were from two prospective studies (EudraCT No. 2007-005809-21; 2012-001353-19) and 41 on a compassionate use study; the median age was 42 and 44, respectively. In both the prospective and compassionate groups, day 28 responses to begelomab were 75% and 61%, respectively, while the CRRs were 11% and 12%. Responses for grade III GvHD were recorded in 83% and 73% of patients, while responses in grade IV GvHD were recorded in 66% and 56% of patients in the two groups, respectively. Interestingly, non-relapse mortality at 6 months was 28% and 38%; and in general, 64%, 56%, 68% responses for skin, liver, and gut stage III–IV GvHD, respectively, were recorded. The 1-year OS was 50% for the prospective studies and 33% for the compassionate use patients. More importantly, of the 28 surviving patients, 12 developed cGvHD, where the severity was mild in five, moderate in five, and severe in two patients. In addition, no adverse events directly attributable to begelomab were reported (30). Begelomab was recently granted Orphan Drug Designations (ODD) by the FDA for the treatment of SR-aGvHD in the USA, EU and Switzerland. While the begelomab effect on aGvHD is an interesting and encouraging finding, there is to date, no clinical trials investigating the effect of begelomab in cGvHD patients. Nonetheless, studies have shown that CD26 may play a role in the development of pulmonary cGvHD, and that treating human umbilical cord blood (HuCB-NOG) transplant mice with the fusion protein caveolin-1-Ig (Cav-Ig), prevents the development of pulmonary cGvHD in these mice by binding to caveolin-1 on the surface of APCs and to CD26 on T cells, thereby, blocking the interaction between CD26 and caveolin-1 (137). Whether begelomab can have a similar effect in pulmonary cGvHD and/or other types of cGvHD remains an open question.

***Infliximab*** is a chimeric monoclonal anti-TNF-α antibody, which has been successfully employed for treating aGvHD (29, 138). For cGvHD, it has been reported that two infusions at 10 mg/Kg i.v. resolved recurrent pericardial effusion related to cGvHD in a pediatric patient (139). Another anti-TNF-α agent, which is a fusion protein with promising signs of ameliorating cGvHD, is ***Etanercept***. Etanercept was administered to 21 patients with steroid-refractory aGvHD (SR-aGvHD) and cGvHD at 25 mg twice weekly for 4 weeks, followed by 25 mg weekly for another month. Overall response rate (ORR) was 52%, with higher efficacy in gastro-intestinal aGvHD (140).

***Alemtuzumab*** is an anti-CD52 mAb, which targets not only lymphocytes including B cells, T cells and NKs, but also DCs (141, 142). Alemtuzumab has already been employed for the treatment of chronic lymphocytic leukemia (107). In 2008, alemtuzumab was reported for the first time as an effective agent in a patient with extensive cutaneous cGvHD, who was already treated unsuccessfully with steroids, cyclosporine-A, sirolimus, tacrolimus, mychophenolate mofetil, infliximab, and rituximab. In this patient, all ulcers and pain were resolved by month 7 of therapy with alemtuzumab, administered at 10 mg/day subcutaneously for six consecutive days every 4 weeks (143). Importantly, not only is alemtuzumab effective in treating cGvHD, but is also able to prevent or lessen the incidence of the disease (144, 145), all of which highlights the significance of this agent in both prophylaxis and treatment of cGvHD.

***Brentuximab Vedotin*** is an anti-CD30 antibody-drug conjugate already employed in Hodgkin’s (146) and in cutaneous lymphomas (147). When used to treat cGvHD, it offered 47% PRs, with 65% of patients able to decrease the steroid dose. Notwithstanding its good efficacy, the toxicity was judged too high, with 41% of patients developing grade 3 or 4 adverse events (in particular severe peripheral neuropathy), thus sustaining the need of larger studies in cGvHD with this drug (148).

***Axatilimab (SNDX-6352)*** is a humanized IgG4 mAb that binds to the colony-stimulating factor 1 receptor (CSF-1R) and inhibits its function. CSF-1R is a receptor for the cytokine CSF-1, which is responsible for the production, differentiation and function of macrophages. By binding to CSF-1R and inhibiting it, axatilimab may be able to disrupt the activities of donor-derived pro-inflammatory macrophages that have been shown to promote cGvHD in the lung and skin (149). Axatilimab is currently being evaluated in a phase I study for its effects in patients with active cGvHD (ClinicalTrials.gov identifier: NCT03604692). Nevertheless, the role of anti-CSF-1R antibodies in cGvHD treatment needs further studies, because it has been reported that axatilimab is also able to reduce resident macrophages, while having no effect on inflammatory monocyte recruitment in models of aGvHD (149).

***Alefacept*** is a full dimeric human leukocyte-function-associated antigen (LFA)-3/IgG1 fusion protein used for treating psoriasis; a disease which is now commonly classified as an autoimmune disease. Alefacept inhibits the activation of T cells by interfering with the CD2 receptor on T cells, and thus blocks T cell proliferation (150–152). Alefacept was demonstrated to be effective for also treating cGvHD, with eight out of 12 patients responding after 2 weeks of treatment. Unfortunately, this response was only temporary, and infections, pericarditis and a squamous cell carcinoma of the lip were observed (108).

***Abatacept***, a soluble fusion protein comprising the extracellular domain of human cytotoxic T-lymphocyte antigen (CTLA)-4 linked to the Fc portion of IgG1 and interferes with the CD28 signaling pathway of T cells by interfering with CD80 and CD86 expressed by antigen-presenting cells (APC). This compound is used for treating the autoimmune disease rheumatoid arthritis, and also seems promising for the treatment of Scl-cGvHD. When administered at 10 mg/kg, it has been reported to be well-tolerated and to offer 44% of PRs and reduction in prednisone usage to half of the responsive patients (123).

### Other Novel Strategies to Target cGvHD Effector Cells

Other novel approaches to target cGvHD effector cells include:

**Ⅰ Blocking cytokines**, such as IL-17 and IL-22, which are responsible for Th17-mediated cGvHD (153, 154), or using low doses IL-2 to induce the expansion of Tregs, which will in turn suppress Tfh cells (155). Tfh cells can promote cGvHD through their support for B cells in GC development, allowing B cell differentiation into plasma cells, leading to the deposition of allo-antibodies into target organs (156).

The possibility of targeting ROCK2, which is able to bind phospho-STAT3 to form a ROCK2/STAT3/JAK2 complex and control the Th17 genesis, was recently reported (157). At the 2018 American society of hematology (ASH) meeting, it was demonstrated that the new ROCK2 inhibitor ***KD025*** is able to reduce levels of IL-21 and IL-17 and to increase the number of Tregs. In a small series of 17 patients who already received three lines of therapy, KD025 induced within 2 months, 65% of responses across all affected organ systems except lung; 82% of them sustained for more than 20 weeks (158).

In addition, high levels of both IL-17 and IL-22 have been reported in the skin of patients with cutaneous cGvHD, which was also attributed to an IL-6-dependent increased secretion of these cytokines by donor CD4^+^ T and IL-22^+^-Th cells (153). The ***anti-IL-22 mAb*** could be a possible option for treating cGvHD, as already demonstrated in murine models for aGvHD, where it reduced TNFα and IFN-γ levels, but at the same time, increased Treg populations (159). Two clinical trials with the anti-IL-22 antibody in aGvHD are now in progress in the USA (ClinicalTrials.gov identifier: NCT02406651, NCT03763318), paving the way for a possible translation into a cGvHD setting.

**ⅠⅠ Blocking CD40L and IL-21R** to prevent Tfh and GC B cell response (37, 160).

**ⅠⅠⅠ Inhibition of the NAD-dependent protein deacetylase sirtuin 1** (sirt1), which regulates different subsets of T cells and is required not only for maintenance of T cell tolerance, but also for promoting Th17 responses (161–164). The important role played by this enzyme in B- and T cell interaction during the development of GvHD was recently highlighted by Daenthanasanmak *et al*. (165). In a murine model, the authors demonstrated that donor T cells lacking sirt1 reduced B cell activation and differentiation but increased Tregs. Moreover, administration of the sirt1 inhibitor ***selisistat (EX 527)*** resulted in reduced Tfh responses along with preventing and treating cGvHD by downregulation of IFN-γ, IL-17, and IL-21, which have all been associated with cGvHD pathogenesis (165);

**IV Inhibition of the Hedgehog (Hh) signaling pathway**. This pathway is involved in the regulation of cellular differentiation during embryonic development (166) and in control of cell proliferation and carcinogenesis (167). The role of Hh signaling in cGvHD was investigated by Zerr et al. (88), where they clearly demonstrated how the Hh pathway plays an important role in the pathophysiology of cGvHD. Based on these preclinical results, ***Vismodegib***, a potent inhibitor of Hh, and already employed for basal cell carcinoma, is currently being evaluated for the treatment of cGvHD (ClinicalTrials.gov identifier: NCT02337517). It has been reported in a very small series that Vismodegib at 150 mg/day induced partial responses in five out of eight patients; nevertheless, half of them stopped treatment due to toxicity (dysgeusia, fatigue, elevated lipase). The median time to response was 103 days and the duration of response was 7.8 months (87). Another Hh inhibitor, ***Sonidegib***, offered 47% of partial response in skin in a small series of 8 patients with Scl-cGvHD; unfortunately, patients reported worsening of quality of life, especially in non-responders (89).

**V Inhibition of NOTCH signaling**. In a murine model of multi-organ cGvHD, it was recently reported that Delta-like ligand 4 (Dll4)-driven NOTCH signaling is essential for the development of cGvHD and that Ab-mediated (***anti-Dll1****/* ***anti-Dll4***) blockade of this pathway prevented and treated cGvHD (168). Indeed, the activation of NOTCH signaling induces an increased production of pro-inflammatory cytokines, decreases Tregs and increases pathogenic GC B cell population through increase in Tfh cells, leading to an increased tissue damage and collage deposition. Inhibition of NOTCH signaling on the other hand, impedes the production of pro-inflammatory cytokines, increases Tregs and inhibits GC formation, hence, preventing target organ damage (168).

**VI Inhibition of the mitogen activated extracellular signal regulated kinases 1 and 2 (MEK1 and MEK2)**, which are important components of the MAPK signaling pathway. In murine models, the RAS/MEK/ERK pathway is activated in naive but not in effector memory T cells (169). Consequently, the MEK inhibitor ***Trametinib*** suppressed GvHD-inducing T cells while sparing antitumor GVL and virus-specific T cells in murine models characterized by skin sclerosis and alopecia (170). Sclerosis and alopecia are both not cGvHD but are high frequency cutaneous manifestations that often occur in cGvHD (171, 172), which makes MEK inhibition relevant to cGvHD.

**VII Inhibition of the PI3K signaling pathway**. PI3Ks are a family of kinases of different classes involved in several cellular processes including metabolism, growth, differentiation, and proliferation. PI3K delta (PI3Kδ) is an isoform preferentially expressed in leukocytes; it seems to control IL-17 production, and its inhibition has been reported to decrease the symptoms of cGvHD in murine models (173, 174). It was shown in a bronchiolites obliterans cGvHD mouse model that PI3Kδ activity in donor T cells is required for the development of cGvHD. In the same study, the authors were able to interestingly demonstrate that the PI3Kδ inhibitor, ***GS‐649443***, was able to treat either BO or Scl-cGvHD by limiting IL-17 production (175). The possibility of testing PI3Kδ inhibitors as a therapeutic strategy for cGvHD is sustained also by the observation that ***Idelalisib***, which is also a PI3K inhibitor, is already employed in relapsed follicular lymphoma and chronic lymphocytic leukemia (176), where it was shown to not increase the onset of cGvHD when administered to patients relapsed after alloHSCT (177).

**VIII Inhibition of Inositol Kinase B**. Inositol triphosphate kinase B (ITPKB) is an enzyme involved in the regulation of calcium intracellular levels, which is fundamental for T cell activation and differentiation. It has been reported that ITPKB inhibition induces apoptosis of activated T cells, and can control T cell–mediated autoimmunity (178). Recently, it was shown in two murine models of cGvHD (Scl and OB) that the ITPKB inhibitor GNF362, significantly improved skin and lung GvHD scores either by reducing M2 macrophage infiltration, or by decreasing the number of IFN-γ and IL-22 producing CD4^+^ T cells, without impairing the graft-versus-tumor effect (179). There is however, to date, no clinical trials with ITPKB inhibitors for the treatment of cGvHD.

## Concluding Remarks and Future Perspective

**Figure 3:**
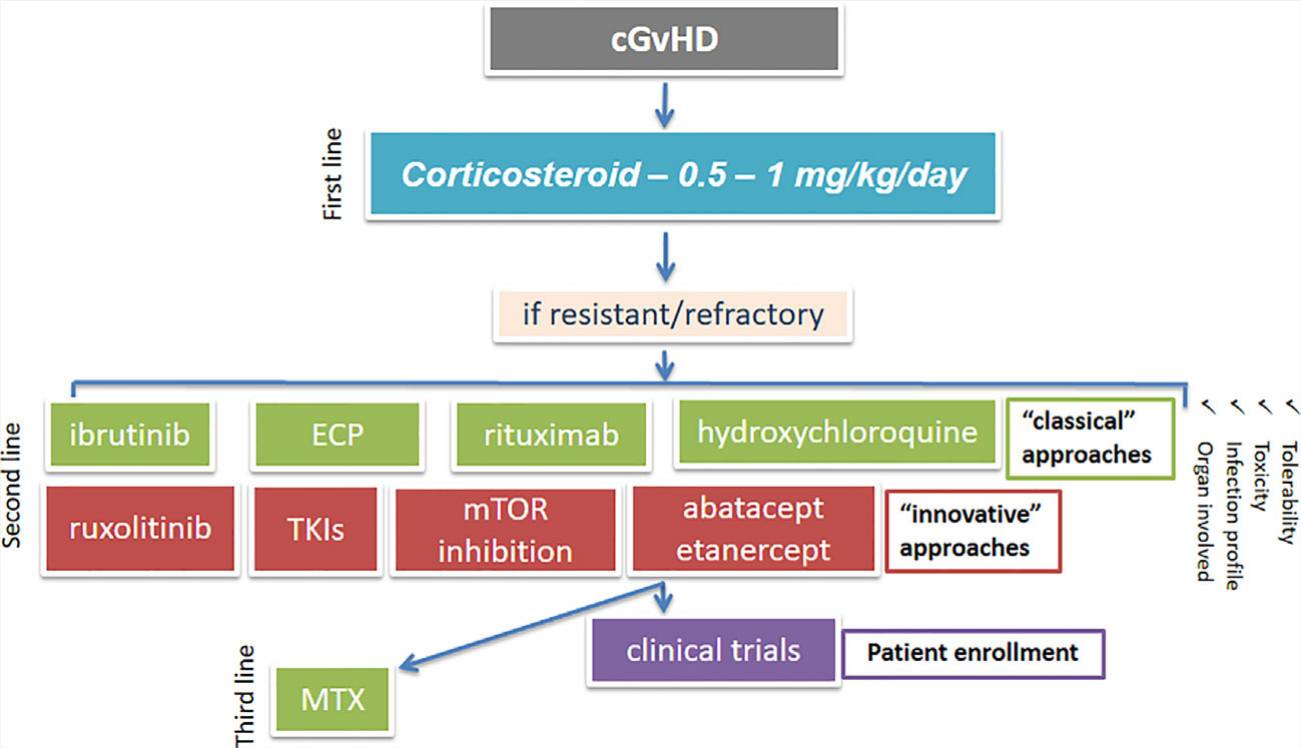
An algorithm showing possible treatment options for cGvHD. cGvHD, chronic graft-versus-host disease; mTOR, mammalian target of rapamycin; ECP, extracorporeal photopheresis; TKI, tyrosine kinase inhibitor; MTX, methotrexate.

We have summarized the more recent advances regarding the preclinical and clinical activities of some newer immunotherapy-based strategies for SR-cGvHD treatment, that include monoclonal anti-B and T cell antibodies, along with TKIs, JAK-, MEK-, proteasome-, and PI3K-inhibitors. An algorithm showing possible second and third-line treatment options for cGvHD is given in **Figure 3**. All these new strategies seem to offer great opportunities for preventing or reversing cGvHD, as they are aimed at repairing/maintaining immune regulation by targeting B cell (responsible for the production of self-reactive antibody complexes) or T cell (responsible for pro-inflammatory and pro-fibrotic cytokine production) signaling and by reducing inflammation, which is fundamental in the pathogenesis of cGvHD (73). However, the biggest drawback for some of these emerging therapies for cGvHD is the available evidence for their tolerability and efficacies, which is frequently from preclinical or clinical trials that are trivial and often conflicting.

Imatinib, dasatinib, nilotinib, ibrutinib, ixazomib, bortezomib, and mAbs (anti-CD20, -CD30, -CD52) are all today commonly employed in the clinical routine for treating acute and chronic leukemias, myeloma and lymphoma. Nevertheless, our knowledge on their efficacies and toxicities in the context of cGvHD is still quite incomplete and many questions remain unanswered; including: What is the tolerability of each of these promising approaches in the setting of cGvHD? What could be the less toxic and, at the same time, the most effective compound? Are the adverse events that are already observed in the treatment of cancers the same when these drugs are employed in patients already allo-transplanted and receiving many concomitant (also immunosuppressive) drugs? Is there an increased risk of secondary malignancies during these new treatments? Is it possible to design an algorithm that could help physicians to choose the most effective drug according to the organ-specific clinical features of cGvHD? Unfortunately, the majority of these questions are still unresolved and to date, the only FDA-approved drug for the treatment of adult patients with SR-cGvHD is ibrutinib, which, as mentioned above, seems to be effective and well tolerated (61). Importantly however, some mAbs, TKIs, and JAK1/2 inhibitors are now in their late stages of evaluation and some are even already employed in the clinics for “compassionate use”.

Perhaps some additional aspects, such as the pharmacogenetic aspects, could help us to design for each patient a “tailored” therapy. For example, it would be incorrect to use imatinib in patients without an effective influx pump or with a high efflux of the drug; in these cases, a TKI such nilotinib, which is superior to imatinib could be advantageous.

We did not summarize options for cGvHD treatment using cell-based therapies, such as mesenchymal stroma cells, regulatory T cells, exosomes, and others, which will be addressed in an additional paper.

Profound knowledge gaps in fully understanding the biology of cGvHD have until now mired the discovery and implementation of effective therapeutic strategies. Nonetheless, we are hopeful that murine models and clinical data, which are already available, will soon be able to help clinicians to control or eradicate cGvHD complications of HSCT, thus, ameliorating the quality of life of transplanted patients.

In this complex scenario, the COST ACTION 17138 “EUROGRAFT” project (see “gvhd.eu” website), in the context of whom this review has been realized, will lead to an improved understanding of the pathogenesis of cGvHD and its associated comorbidities, and will help us to develop a coordinated approach to therapy, *via* a strict international collaboration among experts in this field.

## Author Contributions

NS and SG planned and wrote the manuscript. NS and SG drew the figures. CB, AD, MG, MI, UK, AT, and RZ discussed the content and contributed to writing. All authors contributed to the article and approved the submitted version.

## Funding

This work was supported by COST (European Cooperation in Science and Technology), www.cost.eu—CA17138 EUROGRAFT. AT was supported by the French Government’s Investissement d’Avenir Program, Laboratoire d’Excellence “Milieu Intérieur” Grant ANR-10-LABX-69-01 and by the by the Agence Nationale de la Recherche (Project RANKLthym ANR-19- CE18-0021-02).

## Conflict of Interest

The authors declare that the research was conducted in the absence of any commercial or financial relationships that could be construed as a potential conflict of interest.
